# Supplementary material for: Ectopic Expression of Neurod1 Is Sufficient for Functional Recovery following a Sensory–Motor Cortical Stroke
Source: Biomedicines. 2024 Mar 15;12(3):663. doi: 10.3390/biomedicines12030663 (PMC10968474; doi:10.3390/biomedicines12030663)
Supplement: Supplementary file 1 [file biomedicines-12-00663-s001.zip › biomedicines-2877433-supplementary.pdf]

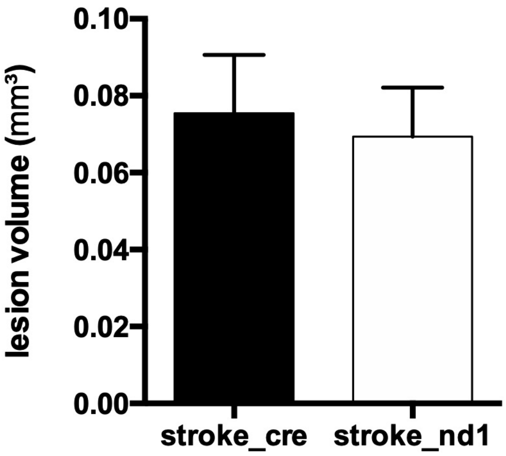

Supplemental Figure S1. Lesion volume. Volume (mm^3) of the lesion was similar between stroke animals that received Neurod1 (stroke_nd1) and Cre (stroke_cre) injections. $p > 0.05$. n = 6–8/group. Data are expressed as mean ± SEM.
